# Hospital Construction

**Published:** 1897-11-27

**Authors:** 


### The Institutional Workshop.

### HOSPITAL CONSTRUCTION.

### OPENING OF THE PARK HOSPITAL.

The Park Hospital at Hither Green, the latest of the long series of fever hospitals built by the Metropolitan Asylums Board, has now been opened for the admission of patients, and the 500 additional beds thus placed at the disposal of the managers will no doubt greatly lessen the strain upon the accommodation which always takes place at this season of the year.

On the eve of the opening, when all was ready for the admission of the patients, we paid a visit of inspection to the institution, and we have no hesitation in saying that, although the cost has no doubt been great, it is most admirably fitted for the treatment of the diseases for the reception of which it is intended. To those whose ideas of hospital matters are derived from the ordinary type of general hospital, such as is to be seen in most of our large towns, the most striking feature of this, or indeed of most of the metropolitan fever hospitals, is the multitude of separate buildings which are dotted over the ground. The total number of beds provided for patients is 548. The site covers 20 acres of ground; there are six miles of drains within this area, 29 miles of water and steam pipes, three miles of roof gutters, and 42 miles of electric wires. Finally, there are 42 separate buildings. To all intents and purposes we have a series of separate isolated hospitals, no one of which will contain more than 46 patients.

Our first criticism shall be directed to the corridors by which these buildings are connected to one another. How much they have cost we of course cannot say, but they form a very prominent feature of the hospital. They certainly are not beautiful, as seen from without, and the question arises, are they useful? It must be understood that they consist of a series of the lightest possible brick arches, carrying a flat roof, that the only protection at the sides is what is given by about three feet high of boarding, all the upper part and all the lower part being absolutely open. In case of wind, or storm, or snow, one might almost as well be entirely out of doors. We entirely agree with the desirability of having a complete disconnection between the different blocks; but when it has once been decided that the corridors are to be so open that waterproofs and umbrellas are necessary in going from place to place, we are inclined to question whether the roofs and arches are not so much money thrown away.

There are several points of interest, and even of novelty, in the pavilions. Each of the scarlet fever pavilions is two storeys in height, each storey containing a ward for 20 beds, one for two beds, and one for one bed. The two storeys are absolutely distinct, *i.e.*, there is no internal staircase going from one ward to the others, each being entered separately from the outside. Now this is very good, as carrying to its extreme limits the principle of separating the patients, as much as possible, in small groups. At the same time it seems to introduce needless difficulties in regard to administration. The intention of the managers is to place the acute cases in the upper ward, and let them pass the period of convalescence downstairs. There are many advantages in this arrangement. The treatment, the diet, and the clothing are all different in the acute and the convalescing stages, and it is very desirable that those who are practically in good and even riotous health should be removed from among those to whom quietness may be all important. Moreover, it is thought, and probably with reason, that the nurse who has gained ascendancy over these patients during their illness will maintain discipline more easily than would a stranger. Hence, instead of having entirely separate convalescent blocks, the patients as they get better are to be removed downstairs, where they will remain under the same charge nurse. This is all very good; but if the same charge nurse is to superintend both wards, it does seem a little hard that she should have to go out of doors in all weathers to get from the one to the other. Clearly the unit in this case is the pavilion, not the ward, and there is no object in breaking it into two. The unit, in fact, consists of an acute ward, a convalescing ward, separation wards, and other appurtenances, such as nurses' duty-rooms, store-rooms for linen, &c., an open-air balcony upstairs on to which beds can be wheeled in suitable weather, and a large airing ground for the convalescing patients directly accessible from the downstairs ward.

In regard to construction, it is worth noting that the bath-rooms and lavatories are separated from the wards by "cut off" corridors, and that the ceiling of this short corridor is lowered, so that on the one hand the separation is the more complete, and on the other an open space is left between this ceiling and the floor above through which air can freely circulate. All the pavilions are raised above the ground level, so that air can circulate freely underneath.

The ventilation of the wards is by means of inlets under every alternate window and under every bed. The latter inlets are formed of bricks perforated with conical orifices—a very useful plan. Those under the windows open into boxes containing hot-water pipes, and then discharge upwards into the wards. Although these boxes are provided with doors for cleaning purposes, we cannot but fear that they will quickly become charged with dust. Even with the doors they are difficult to clean.

The foul air is intended to leave the wards by means of the chimneys, and by flues which are arranged around them. With the air moving at 2½ feet per second, it is calculated that 6,000 cubic feet per patient will be removed per hour. These faieance chimney - stacks, with their Teale grates and hearths, are certainly very attractive, but we fear they must have cost a good deal of mo[illegible]. The same criticism applies to the windows. Some of these, which have been devised by the architect, are admirable; still the question arises whet[illegible]

simpler means would not have been sufficient. If there is a window between every bed and its neighbour, and if the top third and bottom third of every window opens wide, it seems quite unnecessary to complicate matters by providing sashes merely for the sake of opening the middle third. If, however, such opening is wanted, nothing could be better than the windows arranged by Mr. Edwin T. Hall, the architect. In the bath-room and lavatory blocks there are several points of interest. There are no waste-pipes in the bath-rooms; the three baths discharge into a gutter, which leaves the room by a trap. The closet fittings are of the bracket type, being fixed to the walls so that the floor can be swept at one stroke. The arrangement for emptying and cleaning bed-pans is very effectual, and is stated to be much cheaper than some which are in the market. This also has been devised by the architect.

A special device has been arranged, so that, although the patient can bolt the door of the w.c., the nurse can open it from the outside. Some stress was laid upon this, and no doubt it is a very useful contrivance. It reminds us, however, of Lever's Irishman, who, when the bottom came out of the Sedan chair, said he might as well have walked but for the look of the thing; so we fancy if the door can be opened from the outside it might as well have been left unbolted "but for the look of the thing."

These, however, are trifles. The building is a splendid example of the separate pavilion hospital, and we have no doubt that it will be of very great service in the treatment of metropolitan fevers.

The architect is Mr. Edwin T. Hall, F.R.I.B.A., who has evidently given his whole heart to making the hospital as perfect as possible; and the medical superintendent, to whose care this great establishment is entrusted, is Dr. R. A. Birdwood, a man of immense experience in infectious diseases, who has for years past been an able helper to the Asylums Board in its efforts to cope with small-pox, diphtheria, and scarlet fever. For a long time he was general superintendent of the arrangements by which small-pox has practically been swept out of the metropolis; then, when scarlet fever became so prevalent that the authorities had in an emergency to erect a hospital of 500 beds in abou six weeks, Dr. Birdwood undertook the superintendence of this mushroom establishment—a most difficult task; and now he is head of perhaps the greatest fever hospital in the country. Such men are but little known by their fellow citizens; they are to a large extent cut off even from social converse with those around them, they are even debarred from much of the professional honour which is their due, for the Board sets its face against the independent publication by them of the results of their hospital work; yet the debt which the public owes them is immense. They work for years exposed to all the perils of infection, and all that time they give themselves far more absolutely than any other public servants to the work they have to do; yet at the end of their time they get no superannuation except what they themselves have paid for, while, if they die at their posts, their wives and children are cast out unprovided for. Such is public gratitude.

